# Age-associated and therapy-induced alterations in the cellular microenvironment of experimental gliomas

**DOI:** 10.18632/oncotarget.19894

**Published:** 2017-08-03

**Authors:** Hannah Schneider, Birthe Lohmann, Hans-Georg Wirsching, Kathy Hasenbach, Elisabeth J. Rushing, Karl Frei, Martin Pruschy, Ghazaleh Tabatabai, Michael Weller

**Affiliations:** ^1^ Laboratory of Molecular Neuro-Oncology, Department of Neurology, University Hospital and University of Zurich, Zurich, Switzerland; ^2^ Center of Neuroscience, University of Zurich, Zurich, Switzerland; ^3^ Institute of Neuropathology, University Hospital Zurich, Zurich, Switzerland; ^4^ Department of Neurosurgery, University Hospital Zurich, Zurich, Switzerland; ^5^ Laboratory for Molecular Radiobiology, Department of Radiation Oncology, University Hospital Zurich, Zurich, Switzerland

**Keywords:** age, glioblastoma, VEGF, radiotherapy, microenvironment

## Abstract

The poor prognosis associated with advanced age in patients with glioblastoma remains poorly understood. Glioblastoma in the elderly has been particularly associated with vascular endothelial growth factor (VEGF)-dependent angiogenesis, and early uncontrolled studies suggested that the anti-angiogenic agent bevacizumab (BEV), an antibody to VEGF, might be preferentially active in this patient population.

Accordingly, we explored host age-dependent differences in survival and benefit from radiotherapy (RT) or BEV in syngeneic mouse glioma models. Survival was inferior in older mice in the SMA-540 and and less so in SMA-560, but not in the SMA-497 or GL-261 models. Detailed flow cytometric studies revealed increased myeloid and decreased effector T cell population frequencies in SMA-540 tumors of old compared to young mice, but no such difference in the SMA-497 model. Bone marrow transplantation (BMT) from young to old mice had no effect, whereas survival was reduced with BMT from old to young mice. BEV significantly decreased vessel densities in gliomas of old, but not young mice. Accordingly, old, but not young SMA-540 tumor-bearing mice benefited from BEV alone or in combination with RT. End-stage tumors of old BEV- and BEV/RT-treated mice exhibited increased infiltration of T helper and cytotoxic T cells compared to tumors of young mice.

The SMA-540 model may provide a valuable tool to evaluate the influence of host age on glioblastoma progression and treatment response. The biological host factors that modulate glioma growth in old as opposed to young mice remain to be identified.

## INTRODUCTION

Glioblastomas are the most common malignant primary brain tumors. The incidence increases with age, and higher age is an important predictor of poor survival. More than 40% of glioblastoma patients are older than 65 years of age [1]. Yet, it has remained unclear why elderly people develop such tumors more frequently, and more importantly, it remains incompletely understood why elderly glioma patients benefit less from the current treatment options of radiotherapy and concomitant and maintenance temozolomide (TMZ) chemotherapy (TMZ/RT→TMZ) [2, 3]. Isocitrate dehydrogenase (IDH) mutations, which confer a better prognosis, are virtually absent in glioblastomas of the elderly. While this may contribute to differential outcome between younger and older glioblastoma patients, age remains a negative prognostic factor even if an analysis of outcome by age is restricted to patients with IDH wildtype glioblastoma [4]. At the transcriptomic level, age-specific hypermethylation in polycomb group protein target genes and the upregulation of angiogenesis-related genes have been identified, but no fundamental differences compared with tumors from younger patients [5].

Glioblastoma-associated angiogenesis is a hallmark of malignancy and thought to be highly dependent on VEGF. BEV, an antibody to VEGF, has been approved for the treatment of recurrent glioblastomas in various countries. Interestingly, unlike all other treatment modalities previously assessed, early uncontrolled studies indicated that BEV may provide relatively more benefit to elderly patients both in the recurrent [6, 7] and in the newly diagnosed setting [8]. In a retrospective study of recurrent glioblastoma patients treated with BEV without or with chemotherapy, most often irinotecan, a subgroup analysis based on median age (aged ≥ 55 or < 55 years), revealed that there was only a significant difference in PFS and OS between the BEV-treated and control groups in the older age group [6]. In a single agent study of BEV in recurrent glioblastoma, the median PFS for patients aged above the median 53 years was 30 weeks versus a median PFS of 11 weeks for younger patients [7]. Finally, an analysis of a cohort study of newly diagnosed glioblastoma patients exposed to BEV in the first-line setting indicated worse outcome in younger patients [8]. However, subsequent phase III trials that assessed the addition of BEV to TMZ/RT→TMZ in patients with newly diagnosed glioblastoma reported an increase in progression-free survival, but not overall survival [9, 10]. In these controlled studies, there was only a trend for more benefit in the patient population with an unfavourable prognostic profile, that is, elderly patients with tumors lacking O^6^-methylguanine DNA methyltransferase (MGMT) promoter methylation. Yet, it is likely that the elderly patients enrolled in the phase III trials [9, 10] possessed favorable prognostic factors and do not fully reflect the large population of elderly patients with glioblastoma since they were considered eligible for the triple combination of RT, TMZ and BEV in the context of a clinical trial.

There are various hypotheses regarding the molecular mechanisms underlying constitutive or acquired resistance to anti-angiogenic agents such as BEV, e.g., primary or induced up-regulation of compensatory proangiogenic pathways by glioma cells [11]. Recent data also suggest a role of host inflammatory cell infiltration in this regard [12]. Here we explored host age-dependent differences in syngeneic mouse models regarding natural course of disease, benefit from RT or BEV or both, and host responses under these conditions.

## RESULTS

### Age-related survival differences in mouse glioma models

**Figure 1:**
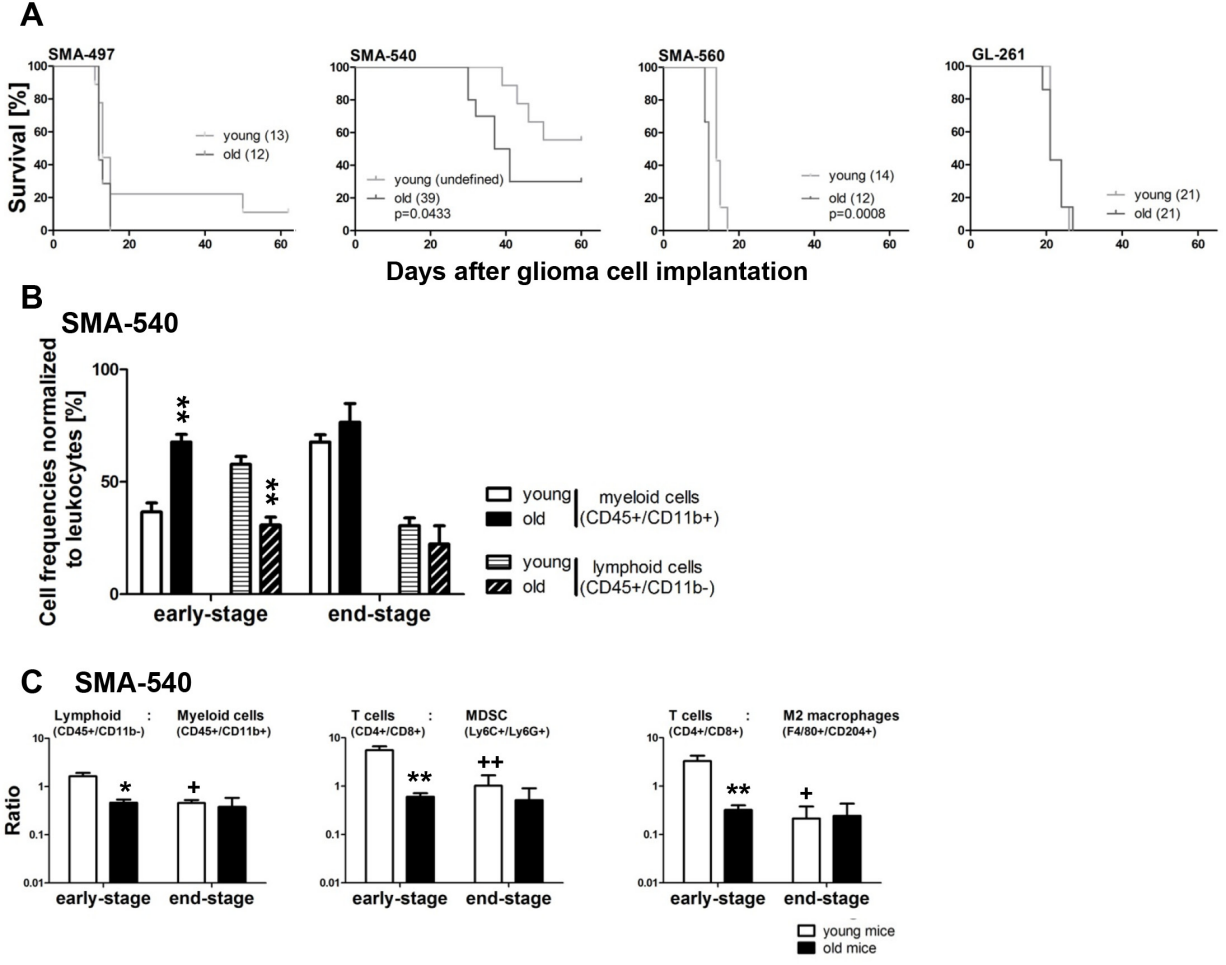
Survival in syngeneic experimental mouse glioma models: modulation by host age **(A)** Glioma cells were implanted in a minimum of seven young (< 3 months) or old (> 8 months) VM/Dk (SMA-497, −540, −560) or C57BL/6 (GL-261) mice. Kaplan-Maier survival curves of glioma-bearing mice are shown for all four syngeneic models. **(B)** Relative frequencies of myeloid (CD45+/CD11b+) and lymphoid (CD45+/CD11b-) cells normalized to total leukocytes in three to five young and old SMA-540 early- and end-stage tumors were analyzed by flow cytometry. Data are expressed as mean and SEM (^**^p<0.01, unpaired Student's t-test, old versus young mice). **(C)** Flow cytometric studies of brain-infiltrating host immune cells in three to five young and old SMA-540 tumor-bearing mice. Different ratios of lymphoid versus myeloid subpopulation frequencies were calculated in early- and end-stage tumors. Data are expressed as mean and SEM (^*^p<0.05, ^**^p<0.01, one-way ANOVA followed by Tukey's post hoc test with a confidence interval of 95%, old versus young mice, ^+^p<0.05, ^++^p<0.01, young end- versus early-stage).

We first explored whether the age of recipient mice modulated the morphology and growth kinetics of syngeneic murine gliomas. For this purpose, we compared mice below 3 months of age with mice above 8 months of age. SMA-497, SMA-560 and GL-261 glioma cell lines were comparably tumorigenic in young and aged mice whereas there was reduced tumorigenicity in young compared to old mice for SMA-540. There was no survival difference in the SMA-497 or GL-261 models between young and old mice. In contrast, survival was inferior in older mice in the SMA-540 and less so in the SMA-560 model (Figure 1A), Table 1. Histological analysis of tumors harvested from mice when the first clinical symptoms occurred (early-stage) revealed densely packed masses of pleomorphic cells with similar tumor volumes in young and old mice in all models (Supplementary Figure 1A, 1E). Proliferation assessed by Ki-67 labeling was similar in tumors derived from young and old animals and restricted to tumor cells and not seen in non-neoplastic cells of the brain (Supplementary Figure 1B, 1E). Microvessel density (MVD), assessed by CD31 labeling, was similar in tumors of all cell lines in all groups, with SMA-560 tumors being the most highly vascularized (Supplementary Figure 1C, 1E) [13]. Based on histological analysis, CD45-positive leukocytes were found in similar numbers with the lowest infiltration density in SMA-560 gliomas (Supplementary Figure 1D, 1E). Tumor-infiltrating leukocyte subpopulations were analyzed in more detail by flow cytometry in syngeneic mouse glioma models of young and old mice with (SMA-540) and without (SMA-497) a survival difference by host age (Figure 1B, 1C, Supplementary Figure 1F-1K). Sham operated mice served as a control that leukocyte infiltration was tumor-related (Supplementary Figure 1L). We observed a relative increase in frequencies, normalized to leukocytes, of myeloid cells, paralleled by a relative decrease in lymphoid cell frequencies, in early-stage SMA-540 tumors of old compared to young mice (Figure 1B). No such differences were detected in SMA-497 gliomas derived from old versus young mice (Supplementary Figure 1H). Of note, only one tumor stage was analyzed in the SMA-497 model due to homogenous onset of neurological symptoms. In the SMA-540 model, there were lower ratios of lymphoid (T cells) versus myeloid (myeloid-derived suppressor cells (MDSC), monocytes, neutrophils, macrophages, M2 macrophages) cell populations in early-stage tumors of old versus young mice. There was also a decrease in this ratio during progression from early- to end-stage disease in young, but not old mice. Further, detailed analysis of subpopulations revealed that ratios of T cells to M2 macrophages or MDSC were lower in old than in young mice in early-stage disease, but also decreased in younger mice in end-stage disease (Figure 1C). A similar analysis of SMA-497 tumors revealed no lymphoid to myeloid differences, but also not in subpopulation cell ratios of tumors of young and old animals (Supplementary Figure 1I).

**Table 1:** Survival in young versus old tumor-bearing mice^1^

	Median survival young mice (days)	Median survival old mice (days)	Young mice versus old mice
**SMA-497**	13	12	p=0.2722
**SMA-540**	undefined	39	^*^p=0.0433
**SMA-560**	14	12	^***^p=0.0008
**GL-261**	21	21	p=0.8365

^1^Comparison of survival curves of Figure 1A (^*^p<0.05, ^***^p<0.001, Gehan-Breslow-Wilcoxon Test, young (< 3 months) versus old (> 8 months) mice).

Next, we interrogated publically available data from The Cancer Genome Atlas (TCGA) to assess differential expression of genes annotated with the gene ontology term biological process: immune_response (GO:0006955) in elderly versus younger glioblastoma patients, defined as age 65 years or older versus younger than 65 years. Established cell surface markers of distinct immune cell populations or master regulator transcription factors that modulate immune responses were not identified by this search (data not shown). However, among genes encoding cytokines and their receptors, we identified lower gene expression values in tissue samples from elderly versus younger patients for interleukin-18 receptor (IL18R1), interleukin-1 receptor (IL1R2), chemokine (C-X-C motif) ligand 6 (CXCL6) and chemokine (C-X-C motif) ligand 13 (CXCL13), whereas the expression of CX3C chemokine receptor 1 (CX3CR1) was higher in glioblastomas of elderly patients (Supplementary Figure 2A). Utilizing median gene expression values to segregate younger glioblastoma patients with high versus low expression of this age-associated immune signature determined overall survival times of 13.1 vs 15.6 months (Supplementary Figure 2B).

### Modulation of glioma growth by the age of the recipient bone marrow (BM)

**Figure 2:**
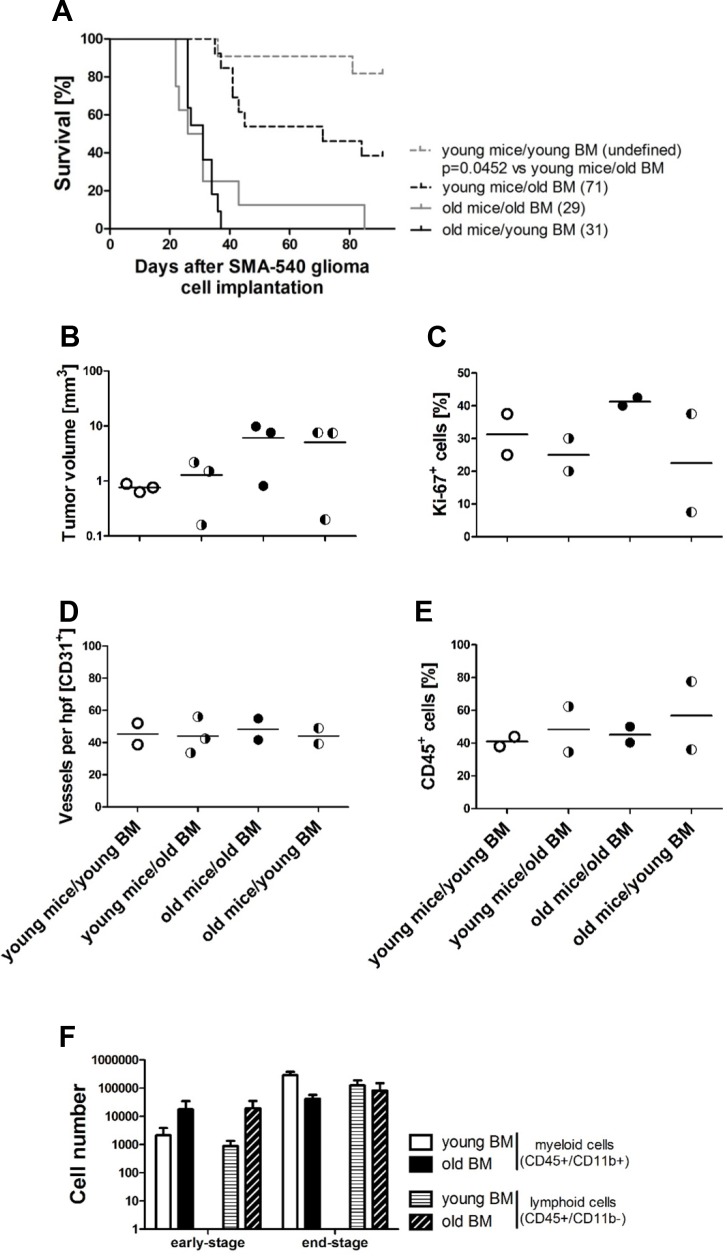
Bone marrow transplantation (BMT) modulates age-dependent survival in the SMA-540 glioma models Glioma cells were implanted in young (< 3 months) or old (> 8 months) female VM/Dk mice. Before surgery, young and old mice were BM-reconstituted receiving young (< 3 months) or old (>8 months) male BM. On day 22, when the first mouse became symptomatic, three tissue samples per group, selected per prerandomization, were harvested for histological analysis and volumetric measurements. **(A)** Kaplan-Maier survival curve of 8-13 mice per group. **(B)** Tumor volumes. **(C)** Proliferation by Ki-67 labeling. **(D)** CD31+ capillaries. **(E)** CD45+ leukocytes. Data are expressed as single values and means of the four ROI per tumor. Groups of tumors were compared (p>0.05, one-way ANOVA followed by Tukey's post hoc test with a confidence interval of 95%, relative to young mice/young BM). **(F)** Absolute numbers of myeloid (CD45+/CD11b+) and lymphoid (CD45+/CD11b-) cells per tumor-bearing hemisphere in young, three to seven BM-reconstituted mice bearing SMA-540 early- and end-stage tumors were analyzed by flow cytometry. Data are expressed as mean and SEM (p>0.05, unpaired Student's t-test, old versus young myeloid cells, p>0.05, old versus young lymphoid cells).

To dissect which component of the aging mouse determined a less favorable outcome in the SMA-540 model, we performed BMT, grafting BM from young mice into old mice, and *vice versa*. The efficacy of BMT was confirmed by detection of the Y chromosome-specific gene zinc finger Y-chromosomal protein (Zfy1) by quantitative real-time polymerase-chain-reaction (qRT-PCR) [14] in female recipient mice and by differential blood count six weeks after BMT (Supplementary Figure 2C, 2D). We noted that BMT from young to old mice did not enhance survival whereas BMT from old to young mice decreased survival (Figure 2A), Table 2. Tumors were harvested for histological analysis on the day when the first mice developed neurological symptoms. No significant increase in tumor volumes was observed in old compared to young mice irrespective of the age of the grafted BM (Figure 2B, Supplementary Figure 2E). There was no difference in tumor cell proliferation (Ki-67) in young versus old animals, irrespective of which BM was transplanted (Figure 2C, Supplementary Figure 2F). MVD and leukocyte infiltration rates were also similar in all groups (Figure 2D, 2E, Supplementary Figure 2G, 2H). Also, we observed no significant differences in the infiltration of absolute numbers of myeloid and lymphoid cells per tumor-bearing hemisphere in the different groups at early-stage of old compared to young mice (Figure 2F).

**Table 2:** Effect of BM transplantation on survival in the SMA-540 model^1^

		Median survival (days)	
**Young mice/young BM**		Undefined	
**Young mice/old BM**		71	
**Old mice/old BM**		29	
**Old mice/young BM**		31	
	**Young mice/old BM**	**Old mice/old BM**	**Old mice/young BM**
**Young mice/young BM**	^*^p=0.0452	^****^p<0.0001	^****^p<0.0001
**Young mice/old BM**		^**^p=0.0011	^****^p< 0.0001
**Old mice/old BM**			p=0.4930

^1^Comparison of survival curves (^*^p<0.05, ^**^p<0.01, ^****^p<0.0001, Gehan-Breslow-Wilcoxon Test) of young (< 3 month) and old (> 8 month) syngeneic SMA-540-glioma-bearing and BM transplanted mice.

### Age-related survival differences in response to RT or anti-VEGF therapy

**Figure 3:**
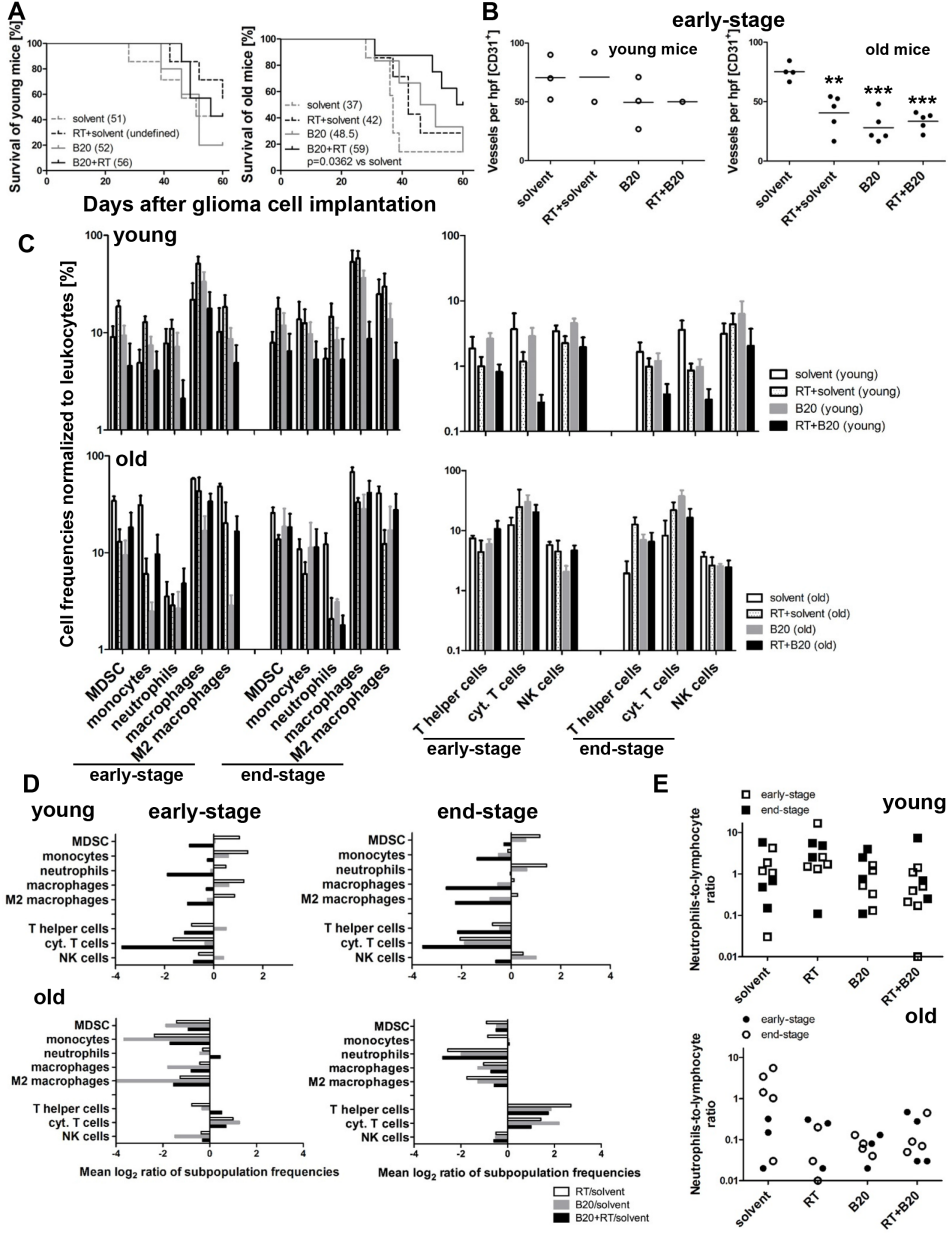
Efficacy of RT and B20 VEGF antibody therapy: modulation by age SMA-540 gliomas were established in young or old mice. The mice were then randomized to either a solvent group or received B20 i.p. (5 mg/kg) twice weekly starting day 10, or a single application of RT (12 Gy) at day 14 to the skull, or both. On day 28, when the first mouse became symptomatic, three to five tumors per group, selected per pre-randomization, were harvested for histological analysis and volumetric measurements. The other five to eight mice per group were monitored for survival. **(A)** Kaplan-Meier survival curves of glioma-bearing young (left) and old mice (right) receiving solvent, RT+solvent, B20 or both. **(B)** Vessel density was determined by counting CD31+ capillaries in four regions of interest (ROI) per tumor. Data are expressed as single values and means of the four ROI per tumor. Groups of tumors were compared (^**^p<0.01, ^***^p<0.001, one-way ANOVA followed by Tukey's post hoc test with a confidence interval of 95%, treatment versus solvent). **(C)** Isolated tumor-infiltrating leukocyte subpopulations of early-stage and end-stage tumors in three to six young (upper row) or old (lower row) mice per group were analysed by multicolor flow cytometry. Frequencies of different myeloid and lymphoid subpopulations normalized to leukocytes. **(D)** Ratios of different tumor-infiltrating leukocyte subpopulations of early-stage (left) and end-stage (right) of solvent versus B20, RT, or BEV/RT were calculated in young (upper row) and old mice (lower row). A value of −2 indicates a reduction to 2^−2^ ≡ 0.25. A value of 2 indicates an increase to 2^2^ ≡ 4. **(E)** NLR of early- and end-stage tumors of young (upper row) and old (lower row) mice.

The SMA-540 model was also chosen to explore potential synergy of RT and VEGF pathway inhibition using the monoclonal anti-mouse VEGF antibody B20-4.1.1 (B20) in young versus old mice. There was no survival benefit from either monotherapy or from combination therapy in young mice. In contrast, median survival was prolonged with RT or BEV, although not significantly, in old tumor-bearing mice. Furthermore, BEV/RT co-treatment was superior to solvent controls in old mice (Figure 3A, Supplementary Figure 3A, Table 3).

**Table 3:** Comparison of survival of SMA-540 tumor-bearing mice treated with RT or B20^1^

Median survival (days)		Young mice		Old mice	
**Solvent**		51		37	
**RT + solvent**		Undefined		42	
**B20**		52		48.5	
**RT + B20**		56		59	
**Young mice**	**RT + solvent**		**B20**		**RT + B20**	
**Solvent**	p=0.3041		p=1.0000		p=0.5488	
**RT + solvent**			p=0.1397		p=0.5398	
**B20**					p=0.3541	
**Old mice**	**RT + solvent**		**B20**		**RT + B20**	
**Solvent**	p=0.1322		p=0.1496		^*^p=0.0362	
**RT + solvent**			p=0.7757		p=0.1590	
B20					p=0.2008	

^1^Comparison of survival curves (^*^p<0.05, Gehan-Breslow-Wilcoxon Test) of young (< 3 months) and old (> 8 months) syngeneic SMA-540-glioma-bearing mice treated with BEV or RT alone or in combination.

Immunohistochemical analysis of early-stage tumors revealed that MVD was significantly decreased in the monotherapy and combination therapy groups only in old mice (Figure 3B, Supplementary Figure 3B). The extent of CD45+ leukocyte infiltration upon mono- and co-treatment was unchanged in early-stage tumors analysed histologically (data not shown). However, flow cytometric analysis of subpopulations of tumor-infiltrating lymphocytes identified increased percentages of T cell populations (T helper cells and cytotoxic T cells) in end-stage tumors from all treatment compared to solvent groups in old mice, whereas we observed a decrease in these populations in young mice (Figure 3C and Supplementary Figure 3C). Accordingly, ratios of lymphocyte subpopulations of treatment versus solvent groups confirmed that treatment caused a relative increase in lymphocyte infiltration in end-stage tumors of old but not young mice (Figure 3D). Furthermore, myeloid subpopulations also differed in old compared to young mice upon treatment. At early tumor stages, anti-VEGF mono-treatment led to significantly reduced MDSC and monocyte infiltration rates; furthermore, anti-VEGF mono- and co-treatment regimens caused a significant reduction of (M2) macrophage infiltration in tumors of old but not young mice (Figure 3C, 3D, Supplementary Figure 3C). Conversely, neutrophil-to-lymphocyte ratios (NLR), as a marker of inflammation [15, 16], revealed that mono- and co-treatment regimens reduced the NLR in old but not young animals (Figure 3E).

## DISCUSSION

There is growing evidence that the interaction of tumor and non-neoplastic cells within the microenvironment of numerous solid tumors, including glioblastoma, determine their growth characteristics and likely also responses to non-surgical treatments like RT or pharmacotherapy [17]. Glioblastoma is characterized by a considerable quantity of infiltrating immune cells mainly consisting of tumor-associated macrophages, which can contribute up to 30% of the tumor mass [18–21]. Many of these host cells may support rather than limit tumor growth in glioblastoma [22–25] and help to maintain an immune suppressive micromilieu. This observation might be of more relevance in supporting tumor growth in brains of elderly patients where local changes in the microenvironment lead to a state resembling chronic inflammation [26].

In the present study, we tried to better understand why old age is such a dominant prognostic factor in glioblastoma and whether the age of the host determines in part the response to therapeutic interventions, notably VEGF antagonism. To address these questions experimentally, we focused on age-related host differences in tumor progression using four syngeneic mouse gliomas as model systems [13]. In addition, we characterized the glioma microenvironment in young and old mice, including changes during RT and anti-VEGF treatment.

Survival was inferior in older mice in two out of four models (SMA-540, SMA-560). An age-dependent survival benefit was not observed in the SMA-497 or GL-261 models. Differences in survival were not explained by altered tumor cell proliferation rates, vessel densities or total leukocyte infiltration rates, at least when assessed histologically at the timepoint when the first mice developed symptoms in each model (Figure 1, Supplementary Figure 1). Detailed flow cytometric analysis in one model with and in another without a survival difference between young and old mice revealed that lymphoid cell frequencies were lower in the SMA-540 early-stage tumors of old mice (Supplementary Figure 1). Furthermore, the ratios of lymphoid to myeloid cells and of total T cells to MDSC and to M2 macrophages were not only lower in old versus young early-stage, but also in young end-stage versus early-stage tumors (Figure 1, Supplementary Figure 1). Such ratios of effector to suppressor cell populations can have a significant impact on tumor growth [27, 28] and might be important factors for a pro- or anti-tumoral effect of the microenvironment [29, 30]. All these differences were seen only in the model with an age-dependent survival (SMA-540), but not in a model without an impact of age on survival (SMA-497).

Decreased survival of old mice might be also related to other factors such as elevated, non-favorable cytokine levels in old animals, e.g. VEGF or the the C-C motif chemokine ligand 2 (CCL2), which increase with normal aging [6, 31]. That systemic rather than local brain-specific aging factors account for poorer survival in old mice was strongly suggested by the unexpected observation that BMT from old to young mice decreased the survival of young recipient mice when challenged with SMA-540 cell implantation (Figure 2, Supplementary Figure 2).

The results of co-treatment studies indicate superior activity of the combination of VEGF inhibition and RT in old mice (Figure 3, Supplementary Figure 3, Table 3). This would be in agreement with two earlier reports indicating that BEV is relatively more effective in elderly than in young patients with recurrent glioblastoma [6, 7]. Yet, such an age effect was less prominent in the large randomized phase III trials in the newly diagnosed setting [9, 10], which may have had a priori selected for better prognosis patients.

Similar to recurrent glioblastomas in the elderly, SMA-540 mouse gliomas in old animals might be more VEGF-dependent than in young mice based on their response to anti-VEGF therapy. MVD was decreased upon B20 treatment regimens only in old mice, consistent with the stronger response to B20 in old mice, indicating also that VEGF-independent pathways may maintain angiogenesis in gliomas of younger mice. Furthermore, flow cytometric studies of tumor-infiltrating immune cell subpopulations revealed that end-stage tumors in old but not young mono- and co-treated mice exhibited increased infiltration of lymphoid cells in relation to myeloid subpopulations, namely T helper and cytotoxic T cells, (Figure 3, Supplementary Figure 3), reflecting an immune activated, anti-tumor microenvironment. This finding was confirmed by lower NLR in the tumors of old compared to young mono- and co-treated mice, reflecting a treatment-related decrease of inflammation, which is considered to be of prognostic significance in several human solid tumors [15, 32], including glioblastoma [16].

Our study highlights the biological host-related and age-induced heterogeneity of murine glioma models. At least for subsets of syngeneic mouse gliomas in old animals, inhibition of VEGF in combination with irradiation results in increased tumor infiltration by lymphoid cells, which in turn might prolong tumor control. Yet, these observations remain preliminary, and further experiments are needed to translate this concept into the human setting of glioblastoma in the elderly where innovative treatment approaches are urgently needed.

## MATERIALS AND METHODS

### Reagents and cell lines

The monoclonal anti-mouse VEGF antibody B20-4.1.1 (B20) was kindly provided by T. R. Schwartz (Genentech, South San Francisco, CA). Murine SMA-497, SMA-540 and SMA-560 glioma cells were kindly provided by D.D. Bigner (Durham, NC). GL-261 cells were received from the National Cancer Institute (Frederick, MD). These cell lines have been characterized extensively in our laboratory [13] and are commonly cultured as adherent monolayers in Dulbecco's modified Eagle medium (DMEM) (Gibco, Life Technologies, Zug, Switzerland) supplemented with 10% heat-inactivated fetal calf serum (FCS) (Biochrom KG, Zug, Switzerland) and 2 mM glutamine (Biochrom KG).

### Animal experiments

C57BL/6 mice were purchased from Charles River Laboratories (Sulzfeld, Germany). VM/Dk mice were obtained from in house breeding. 5′000 SMA-497, SMA-540 and SMA-560 or 20′000 GL-261 cells in a volume of 2 μl of phosphate-buffered saline (PBS) (Gibco, Life Technologies) were injected into the right striatum of young mice (aged < 3 months) or old mice (aged > 8 months) anesthetized and placed in a stereotaxic fixation device (Stoelting, Wood Dale, IL). A burr hole was drilled in the skull 2 mm lateral and 1 mm posterior to the bregma. The needle of a Hamilton syringe (Bonaduz, Switzerland) was introduced to a depth of 3 mm. The mice employed had body weights > 20 g. Using a customized shielding device, mice were given a strictly locoregional (whole brain) radiotherapy of 12 Gy with 200 kV X-Ray unit at 100 cGy/min whereas systemic treatment was performed by i.p. injections of B20 (5 mg/kg body weight 2 times per week) or PBS. The mice were observed daily and euthanized when neurological symptoms developed or at defined time points for histological or flow cytometric analysis as indicated. Three to five mice per group were commonly euthanized using a pre-randomization list when any mouse in the experiment became symptomatic in order to perform histological or flow cytometric studies to assess tumor growth and tumor immune cell infiltration at an early stage. The remaining five to ten mice were euthanized when displaying neurological symptoms to obtain survival or flow cytometric data (end-stage). Where indicated, mice brains were explanted for snap-frozen samples for histology and immunohistochemistry. Accordingly, brains were collected upon euthanization, embedded in cryomoulds in Shandon Cytochrome yellow (Thermo Scientific, Waltham, MA) and frozen in liquid nitrogen. Tumor incidences and sizes were determined using hematoxylin and eosin stained 8 μm thick cryosections using a Microm HM560 (Microchom HM560, Thermo Scientific).

### Generation of BM–chimeric mice

For BM chimeras, male donor VM/Dk mice were euthanized. BM cells from the femurs and tibias were isolated by flushing with PBS containing 2% FCS and 1% penicillin/streptomycin (Invitrogen, Life Technologies, Zug, Switzerland) and filtered through a 70 μM mesh to remove bone spicules or muscle. BM cells (10^6^/ml) were incubated in Hematopoietic Stem Cell Media (Sigma, St. Louis, MO) plus 10% FCS, 1% penicillin/streptomycin (Invitrogen), 20 ng/ml mIL-3, 50 ng/ml mIL-6 and 50 ng/ml murine stem cell factor (mSCF) (Peprotech, London, UK) for up to four days at 37°C with 5% CO_2_ in a humidified chamber. After incubation, BM cells were injected intravenously into congenic recipient male and female mice (6-week-old, lethally myeloablatively irradiated with 10.5 Gy 1-2 hours earlier). Blood chimerism was tested by a qRT-PCR-based technique [14] in female recipient mice. Briefly, genomic DNA was isolated from peripheral blood samples and the Y chromosome specific gene, Zfy1, was amplified. Additionally, differential blood counts of BM-reconstituted or only lethally irradiated, non-transplanted and wild-type (WT) mice were obtained.

### Histology and immunohistochemistry

Primary antibodies were monoclonal rat anti-CD3 (BD Pharmingen, Allschwil, Switzerland, 555273, 1:100), monoclonal rat anti-CD31 (BD Pharmingen, BD550274, 1:50), monoclonal rat anti-CD45 (Biolegend, San Diego, CA, 103102, 1:1000), and polyclonal rabbit anti-Ki67 (Epitomics, Burlingame, CA, 4203-1, 1:100). For conventional histology, 8 μm cryosections were stained with haematoxylin and eosin. For immunohistochemistry, cryosections were pretreated with 1% H_2_O_2_ and blocked in 10% rabbit serum or blocking solution (Candor Biosciences, Wangen, Germany). Biotinylated secondary antibodies, streptavidin and diaminobenzidine were obtained from Dako (Baar, Switzerland). Histofine Simple Stain Mouse MAX PO secondary-labelled antibody system was obtained from Nichirei (Tokyo, Japan). Secondary antibodies were used according to standard procedures. For each experiment, three randomly selected animals per group were euthanized and brains were harvested on the day the first animal developed neurological symptoms.

Immunohistological scores for proliferating cells (Ki-67), blood vessels (CD31), leukocytes (CD45) and T cells (CD3) were obtained from 4 regions of interest (ROI) from 2-3 tumors per group. Within tumor tissue, percentages of CD3-, CD45- and Ki-67-positive cells were determined. MVD was calculated by counting CD31+ capillaries. Tumor volumes were calculated using an approximation based on ellipsoid geometric primitive [33].

### Flow cytometry

The following antibodies were used for flow cytometric analysis: monoclonal rat anti-CD4 (clone Gk1.5, BD, 563232, 1:100), monoclonal rat anti-CD8 (clone 53-6.7, BD, 563332, 1:100), monoclonal rat anti-CD11b (clone M1/70; BD, 557657, 1:100), monoclonal rat anti-CD45 (clone 30-F11, BioLegend, 103126, 1:400), monoclonal rat anti-CD204 (Lifespan Biosciences, Seattle, WA, LS-C130606, 1:50), monoclonal rat anti-F4/80 (clone Cl:A3-1, AbD Serotec, Kidlington, UK, MCA497PET, 1:50), monoclonal rat anti-Ly6C (clone AL-21, BD, 563011, 1:400), monoclonal rat anti-Ly6G (clone 1A8, BD, 560602, 1:100) and monoclonal mouse anti-NK1.1 (clone PK136, BD, 560515, 1:50). Zombie Aqua fixable viability kit (BioLegend) was used to exclude dead cells. Doublets were excluded based on SSC-A/SSC-H.

Flow cytometry of brain-infiltrating leukocytes was performed as described [34]. Briefly, mice were anaesthesized and then cardially perfused using ice-cold PBS. Subsequently, tumor bearing hemispheres were dissected removing the olfactory bulb and cerebellum, cut into small pieces and incubated with DNaseI (0.5 mg/ml, Sigma) and collagenase D (0.4 mg/ml, Roche, Rotkreuz, Switzerland) for 30-45 min at 37°C. Thereafter, the tissue was homogenized, filtered through a 100-μm nylon filter (BD) and washed with PBS. Following centrifugation at 350 g for 5 min at 4°C, the pellet was resuspended in 30% Percoll (Sigma) and centrifuged again at 15,000 g for 30 min at 4°C. The myelin topping was removed and the remaining solution containing brain-infiltrating immune cells was collected and washed with PBS. Flow cytometry was performed according to standard protocols followed by analysis on a LSR II Fortessa (BD). Data analysis was performed using FlowJo Version 10.0.7 (Tree Star).

### TCGA analyses

The TCGA GBM dataset was accessed utilizing cancer browser (https://genome-cancer.ucsc.edu). Illumina HiSeq percentile normalized gene expression data of samples derived from newly diagnosed, non-glioma CpG island methylator phenotype (G-CIMP) glioblastoma samples were analyzed for differential expression of any genes annotated with the gene ontology term biological processs: immune_response (GO:0006955).

### Statistical analysis

Statistical analysis was done using GraphPad Prism 5.0 software. Survival analysis for the *in vivo* studies and the TCGA dataset were done using the Log-rank (Mantel-Cox) test and the Gehan-Breslow-Wilcoxon test. Statistical significance of immunohistochemical, flow cytometry and gene expression data was tested using the unpaired and paired Student t-test or one-way ANOVA with Tukey's post hoc test for multiple analysis. A p value below 0.05 was considered significant.

## SUPPLEMENTARY FIGURES


