# Supplementary material for: Age-associated and therapy-induced alterations in the cellular microenvironment of experimental gliomas
Source: Oncotarget. 2017 Aug 3;8(50):87124–35. doi: 10.18632/oncotarget.19894 (PMC5675620; doi:10.18632/oncotarget.19894)
Supplement: Supplementary file 1 [file oncotarget-08-87124-s001.pdf]

Age-associated and therapy-induced alterations in the cellular microenvironment of experimental gliomas

SUPPLEMENTARY MATERIALS

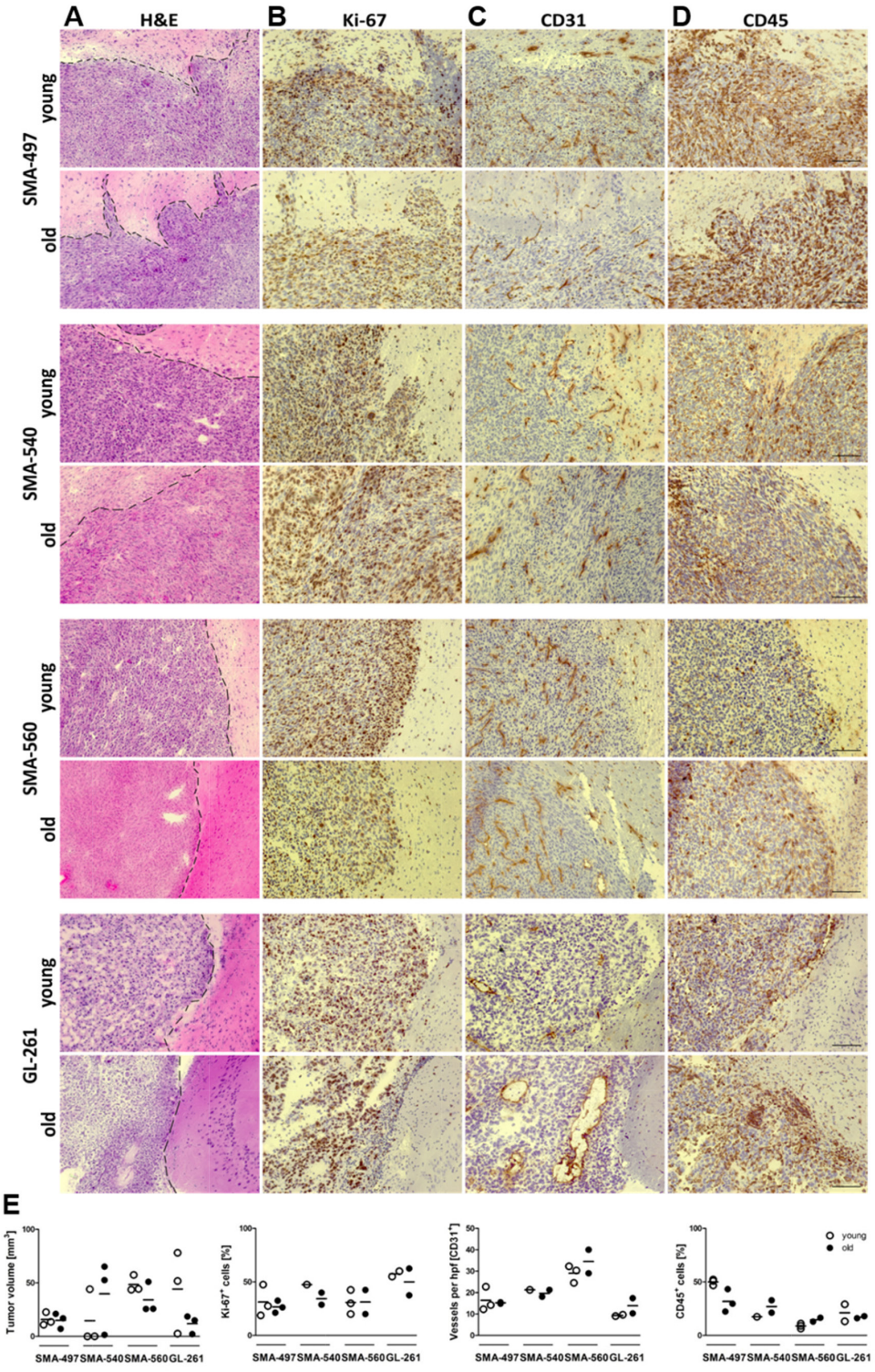

(*Continued*)

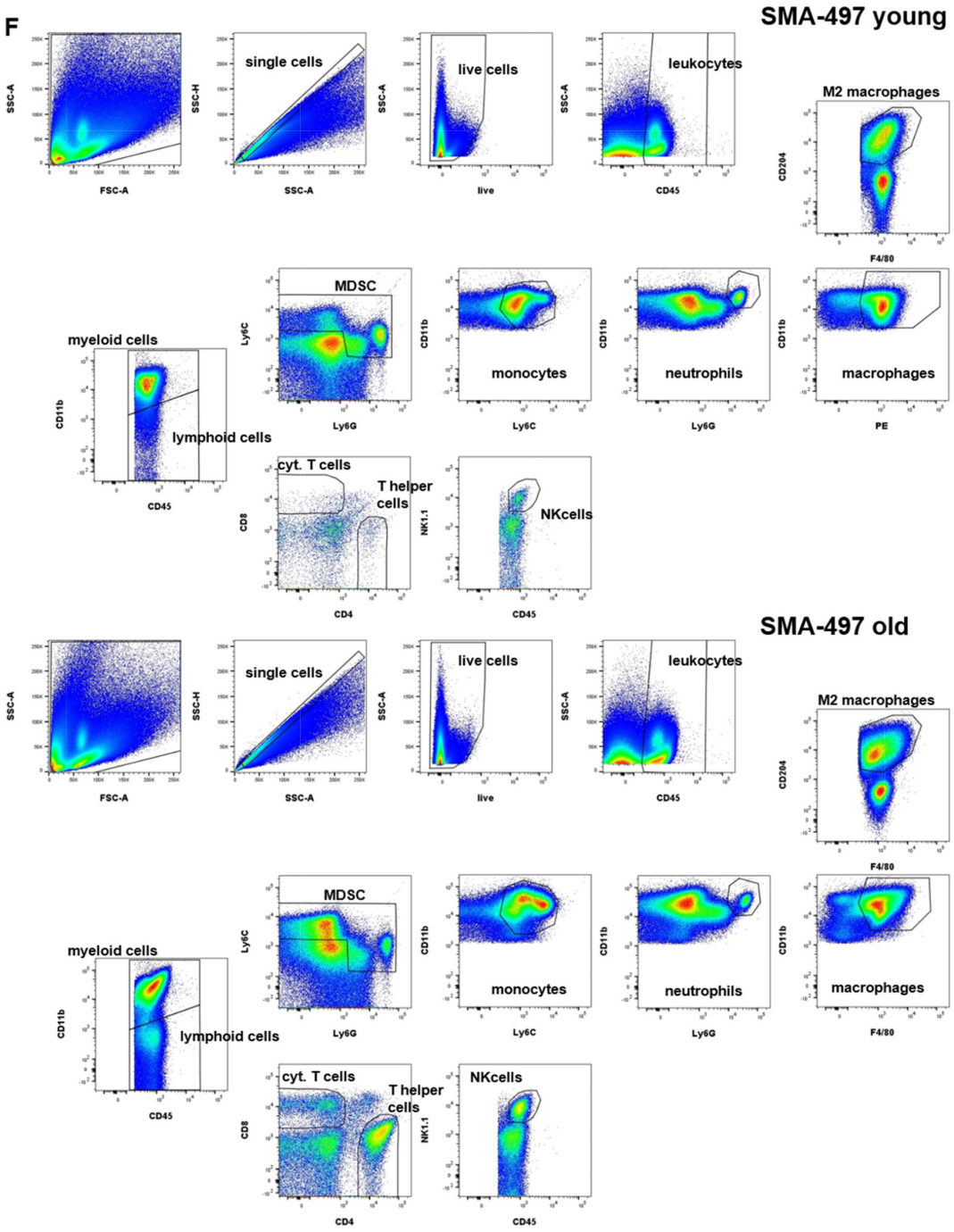

Supplementary Figure 1 (*Continued*): Analysis of the morphology and of the cellular microenvironment of syngeneic experimental mouse gliomas. (A-D) Glioma cells were implanted in young (< 3 months) or old (> 8 months) VM/Dk (SMA-497, SMA-540, SMA-560) or C57BL/6 (GL-261) mice. Three tissue samples per group and model were harvested for histological analysis when the first mouse in any model became symptomatic: day 11 in SMA-497, day 30 in SMA-540, day 12 in SMA-560 and day 19 in GL-261. Tumor morphology was assessed by H&E staining (A), proliferation by Ki-67 labeling (B), blood vessels by CD31 staining (C), and leukocyte infiltration by CD45 staining (D). Light microscopy images are shown at 10x magnification and size bars correspond to 100 µm. **(E)** Tumor volumes were quantified (left). Proliferation was analysed by determining the percentage of Ki-67-positive tumor cell nuclei (middle left). Vessel density was determined by counting CD31+ capillaries in four ROI per tumor (middle right). Percentage of tumor-infiltrating CD45+ leukocytes versus all cells (right). Data are expressed as means of the four ROI per tumor. Groups of tumors were compared (one-way ANOVA followed by Tukey's post hoc test with a confidence interval of 95%).

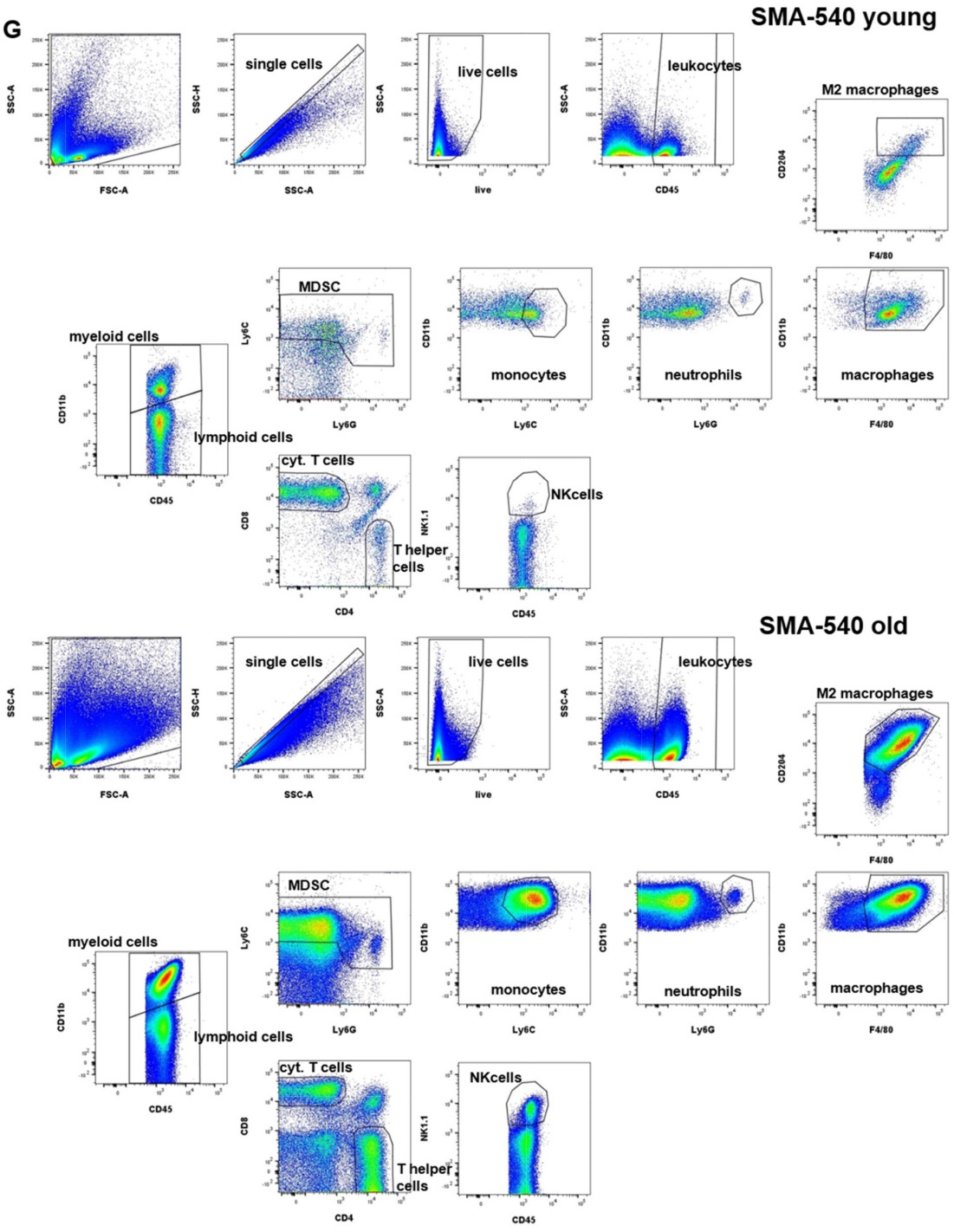

Supplementary Figure 1 (*Continued*): (F, G) Isolated tumor-infiltrating leukocyte subpopulations of tumors were analysed by multicolor flow cytometry. General gating strategy of representative SMA-497 (F) or SMA-540 (G) tumor samples of young (upper panel) or old mice (lower panel): First, forward scatter (FSC-A, log) and side scatter (SSC-A, log) were plotted and all cells were gated. Next, cells were gated for single cells (SSC-A vs. SSC-H), for the uptake of the Live/Dead Aqua stain to determine live versus dead cells (AmCyan vs. SSC-A) and for the expression of CD45 (leukocytes). The leukocyte gate was further analysed for the expression of CD11b+ (myeloid) and CD11b- cells (lymphoid). Different myeloid and lymphoid subpopulations were further gated: from the CD11b+ myeloid cell fraction, Ly6G+/Ly6C+ (MDSC), Ly6C+/Ly6G- (monocytes), Ly6G+/Ly6C- (neutrophils), F4/80+ (macrophages) and F4/80+/CD204+ (M2 macrophages) were identified. CD4+ (T helper cells), CD8+ (cytotoxic T cells) or NK1.1+ (NK cells) surface expression was determined from the lymphoid cell gated population.

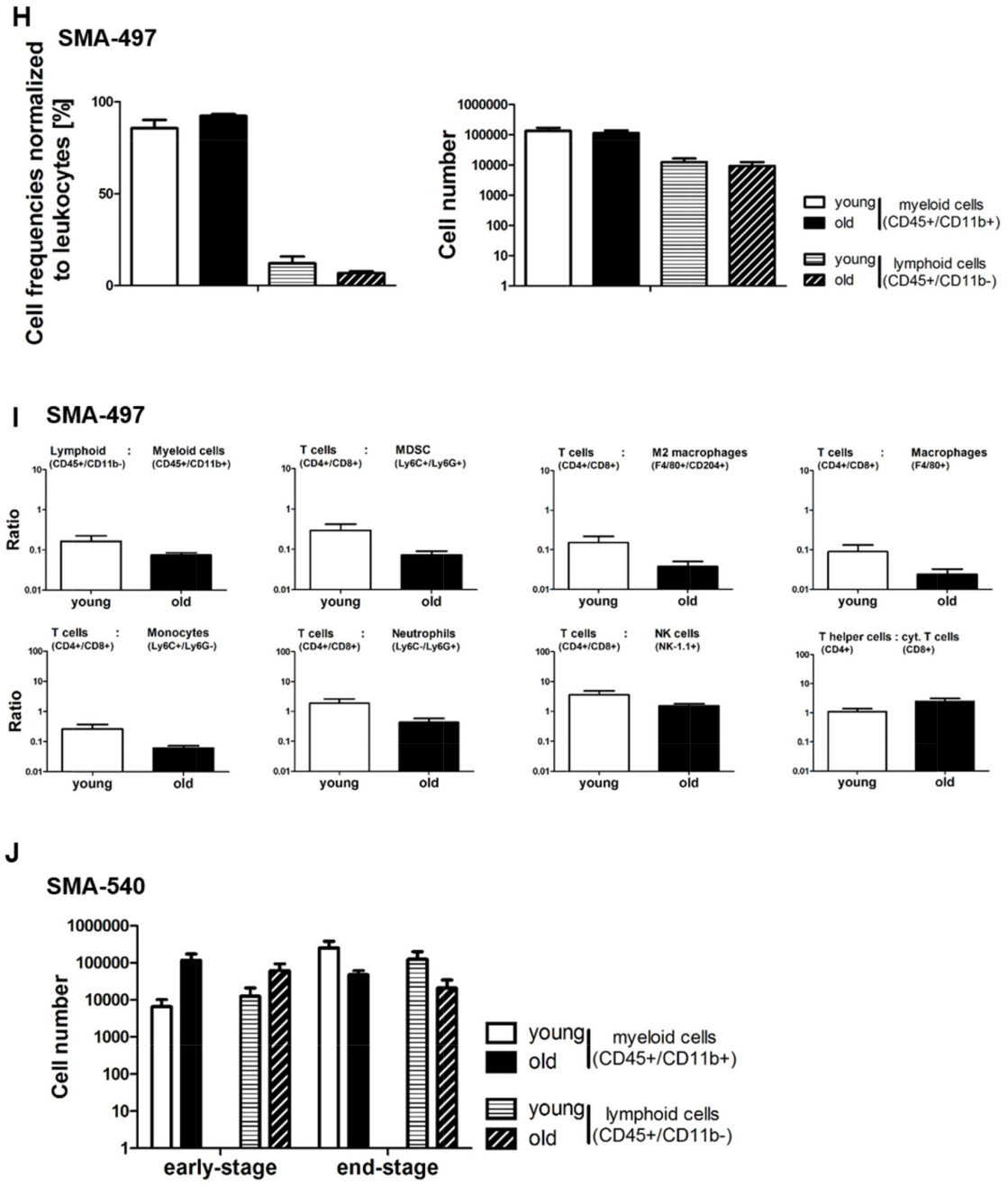

Supplementary Figure 1 (*Continued*): (H) Relative frequencies of myeloid and lymphoid cells normalized to total leukocytes in young and old SMA-497 tumors (left graphs) were analyzed by flow cytometry. Absolute numbers of myeloid (CD45+/CD11b+) and lymphoid (CD45+/CD11b-) cells per tumor-bearing hemisphere in young and old SMA-497 tumors (right graphs). Data are expressed as mean and SEM ($p>0.05$, unpaired Student's t-test, old versus young mice for myeloid cellsor lymphoid cells). **(I)** Flow cytometric studies of brain-infiltrating host immune cells of young and old SMA-497 tumor-bearing mice. Different ratios of lymphoid versus myeloid subpopulation frequencies were calculated. Data are expressed as mean and SEM ($p>0.05$, unpaired Student's t-test, old versus young mice). **(J)** Absolute numbers of myeloid (CD45+/CD11b+) and lymphoid (CD45+/CD11b-) cells per tumor-bearing hemisphere in three to five young and old SMA-540 early- and end-stage tumors were analyzed by flow cytometry. Data are expressed as mean and SEM ($p>0.05$, unpaired Student's t-test, old versus young mice).

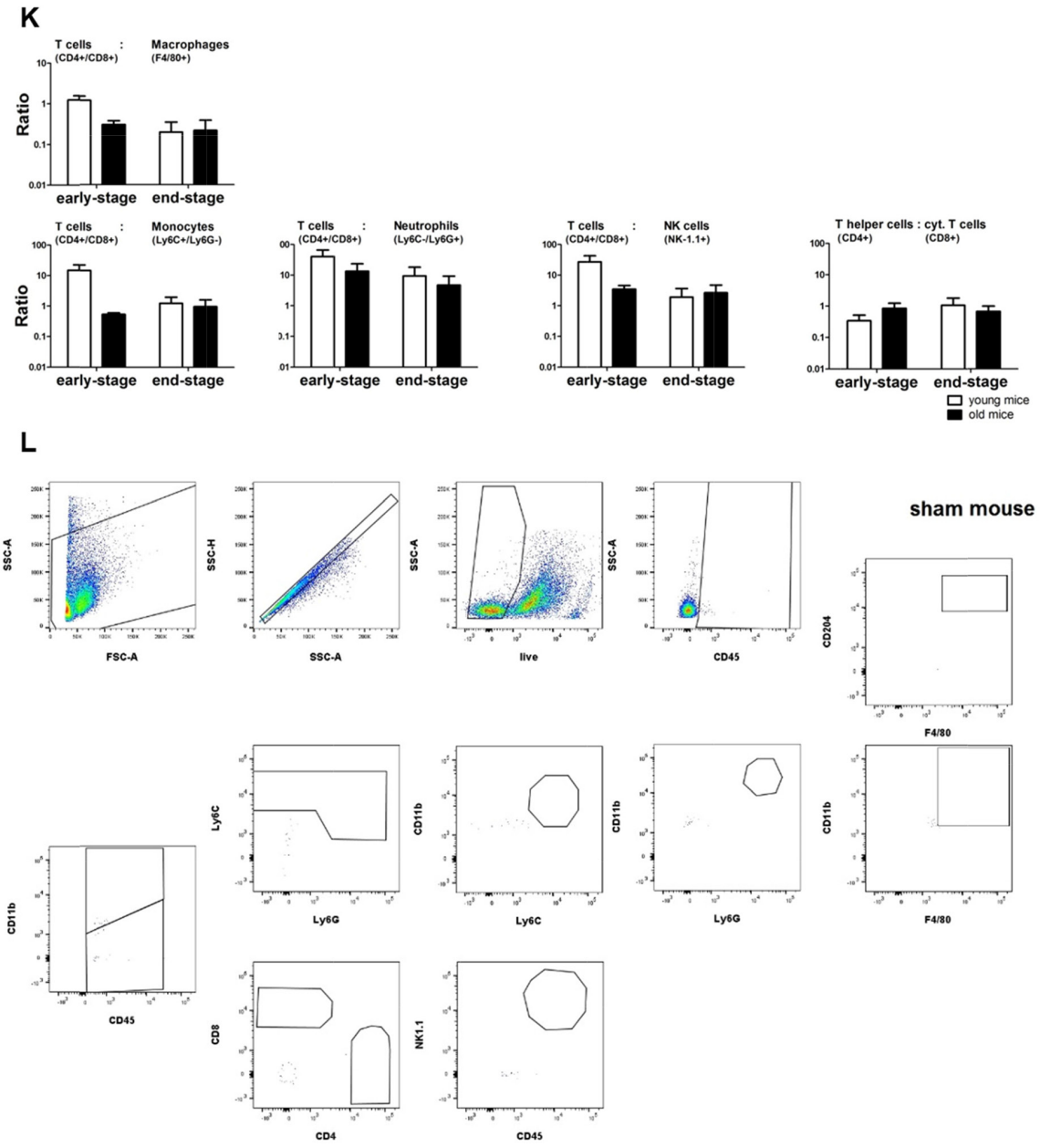

Supplementary Figure 1 (*Continued*): (K) Flow cytometric studies of brain-infiltrating host immune cells in three to five young and old SMA-540 tumor-bearing mice. Different ratios of lymphoid versus myeloid subpopulation frequencies were calculated in early- and end-stage tumors. Data are expressed as mean and SEM ($p>0.05$, one-way ANOVA followed by Tukey's post hoc test with a confidence interval of 95%, old versus young mice or young end- versus early-stage). **(L)** Isolated brain-infiltrating leukocyte subpopulations of the right hemisphere of a sham-operated mouse were analysed by multicolor flow cytometry as also shown in F and G as a control.

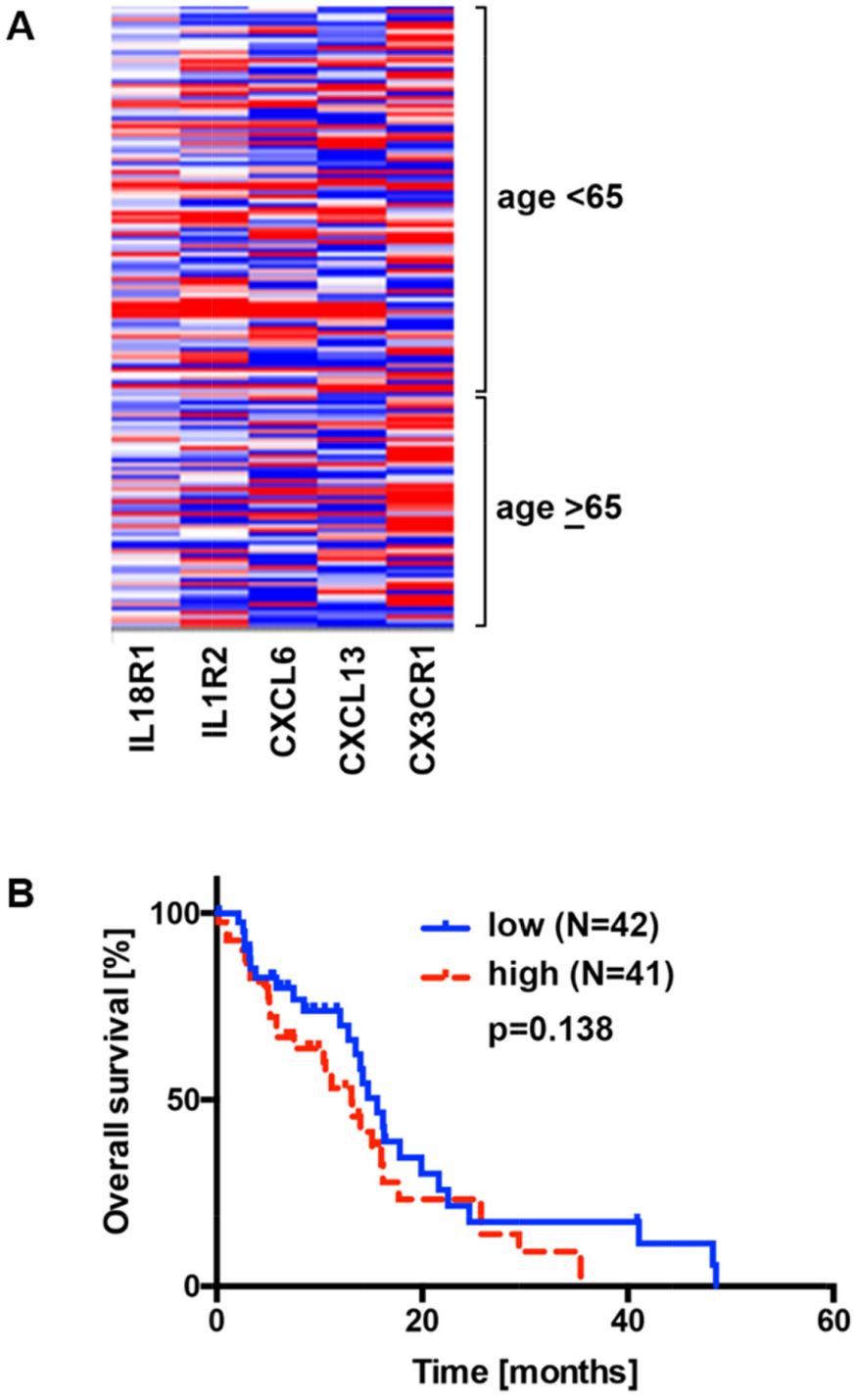

Supplementary Figure 2: TCGA analysis and morphology and outcome in bone marrow (BM)-reconstituted syngeneic mouse glioma models. Association of an age-associated immune geneset with overall survival. **(A)** Cytokines and cytokine receptors that are differentially expressed in glioblastoma patients aged >65 years versus <65 years are depicted. Red versus blue indicate high versus low gene expression. The publically available TCGA glioblastoma Illumina HiSeq percentile normalized geneset was accessed utilizing the cancer browser webtool (2-sided Student's t-test $p<0.05$). **(B)** Median age-associated immune geneset expression was utilized as a cut-off to segregate younger glioblastoma patients with low versus high immune geneset expression for overall survival analyses (hazard ratio 1.47, 95% confidence interval 0.89-2.57, log rank p=0.138). C-H. Glioma cells were implanted in young (< 3 months) or old (> 8 months) VM/Dk mice. Before surgery, young and old mice were BM-reconstituted receiving young (< 3 months) or old (>8 months) male BM.

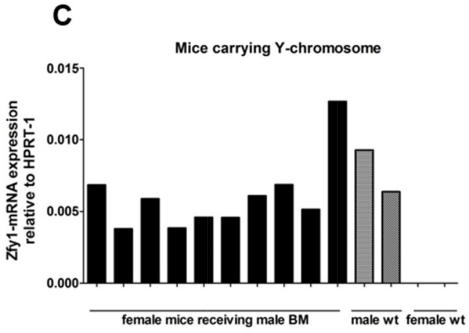

D

Groups	RT only (after 8-10 days)	BM reconstituted mice (after 45 days)	WT (after 45 days)
Result differential blood count	very low leukocyte number	physiological leukocyte numbers, hemoglobin and hematocrit values	

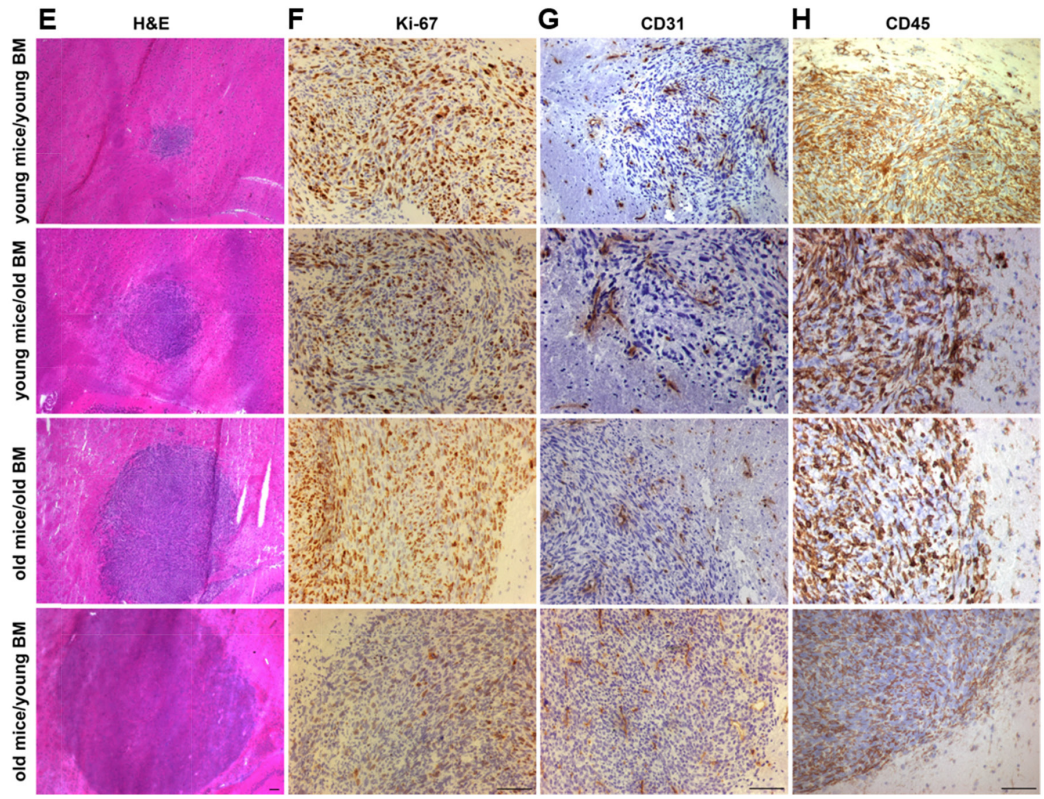

Supplementary Figure 2 (*Continued*): (C) BM cells from male mice were transplanted into whole body-irradiated female hosts. Peripheral blood chimerism at 6 weeks post transplantation were estimated by percentage of male DNA in female recipients by quantitative real-time polymerase-chain-reaction (qRT-PCR). **(D)** Results of differential blood count of whole body-irradiated, bone marrow reconstituted and WT mice. **(E-H)**. On day 22, when the first mouse became symptomatic, three prerandomized tissue samples per group were harvested for histological analysis and volumetric measurements. The morphology of SMA-540 gliomas was assessed by H&E staining (E), proliferation by Ki-67 labeling (F), blood vessels by CD31 staining (G), and leukocyte infiltration by CD45 staining (H). Light microscopy images are shown at 2.5x (C) and 10x (E-H) magnification and size bars correspond to 100 μm.

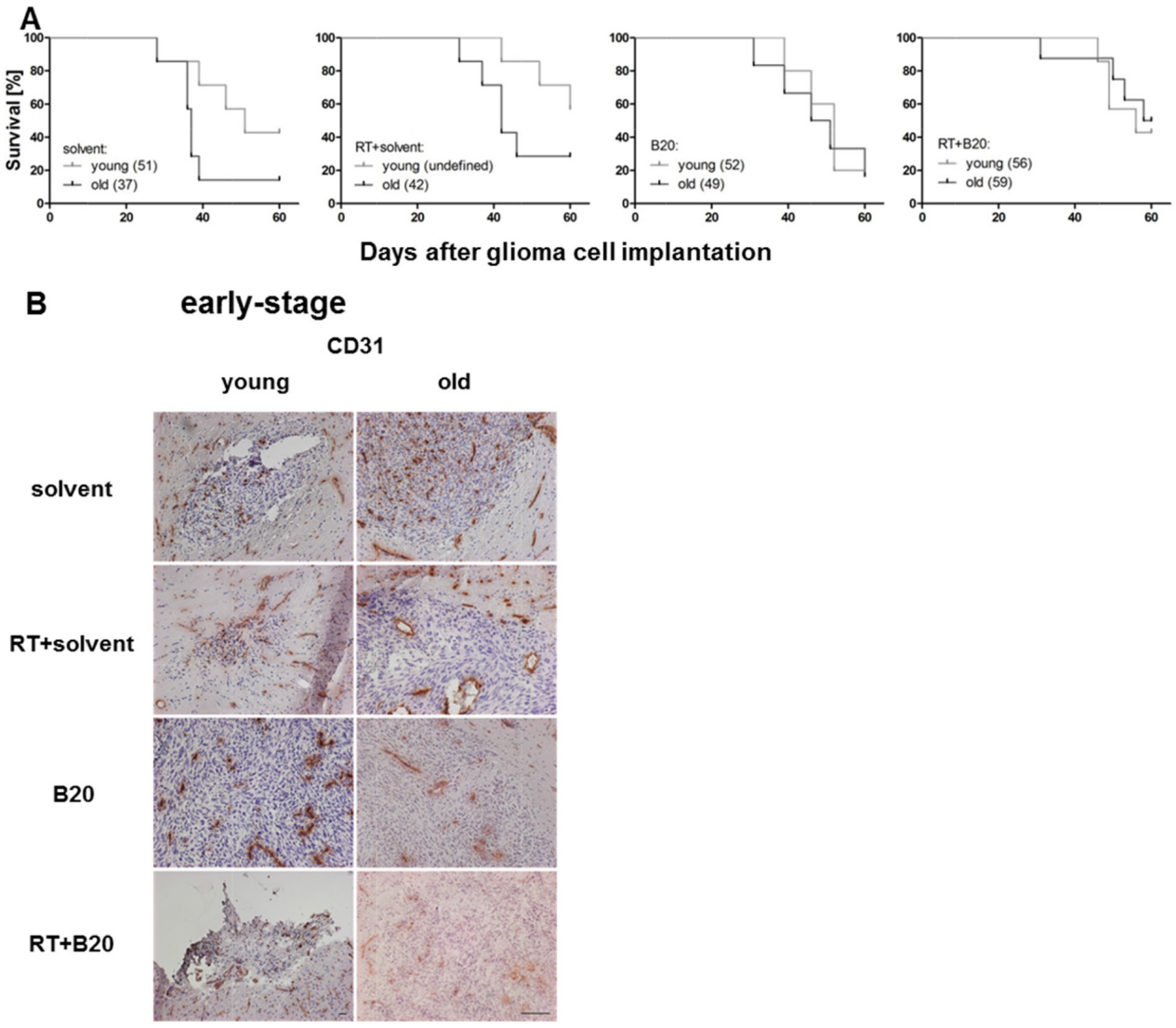

Supplementary Figure 3: Efficacy of radiotherapy (RT) and vascular endothelial growth factor (VEGF) antibody therapy: modulation by age. SMA-540 gliomas were established in young or old mice. The mice were then randomized to a solvent group or to receive B20 i.p. (5 mg/kg) twice weekly starting day 10, or a single application of RT (12 Gy) at day 14 to the skull, or both. **(A)** Kaplan-Meier survival curves of glioma-bearing young versus old mice receiving solvent (left), RT+solvent (second left), B20 (second right) or both (right). **(B)** Blood vessels were identified by CD31 staining. Light microscopy images are shown at 10x magnification and size bars correspond to 100 μm.

C

upregulation (upper versus left condition)
downregulation (upper versus left condition)

		young			old		
		RT+solvent	B20	RT+B20	RT+solvent	B20	RT+B20
		**MDSC**			**MDSC**		
early-stage	solvent	*p=0.0344			*p=0.0225	*p=0.0106	
	RT+solvent		*p=0.035	**p=0.0096			
	B20						
end-stage	solvent						
	RT+solvent						
	B20						
		**monocytes**			**monocytes**		
early-stage	solvent	*p=0.0152			*p=0.0387	*p=0.0217	
	RT+solvent			*p=0.0204			
	B20						
end-stage	solvent						
	RT+solvent						
	B20						
		**neutrophils**			**neutrophils**		
early-stage	solvent						
	RT+solvent			**p=0.0067			
	B20						
end-stage	solvent						*p=0.0437
	RT+solvent						
	B20						*p=0.0423
		**macrophages**			**macrophages**		
early-stage	solvent					**p=0.0044	*p=0.0317
	RT+solvent			*p=0.0246			
	B20						
end-stage	solvent				*p=0.0221	*p=0.0243	
	RT+solvent			*p=0.0146			
	B20			*p=0.0248			
		**M2 macrophages**			**M2 macrophages**		
early-stage	solvent					***p=0.0002	*p=0.0166
	RT+solvent			*p=0.0422			
	B20						
end-stage	solvent				*p=0.0339		
	RT+solvent						
	B20						

		RT+solvent	B20	RT+B20	RT+solvent	B20	RT+B20
		**T helper cells**			**T helper cells**		
early-stage	solvent						
	RT+solvent		*p=0.0463				
	B20			**p=0.0090			
end-stage	solvent				*p=0.0169	*p=0.0373	
	RT+solvent						
	B20						
		**cyt. T cells**			**cyt. T cells**		
early-stage	solvent						
	RT+solvent			*p=0.0473			
	B20			*p=0.0105			
end-stage	solvent					*p=0.0311	
	RT+solvent						
	B20						
		**NK cells**			**NK cells**		
early-stage	solvent					*p=0.0139	
	RT+solvent						
	B20						
end-stage	solvent						
	RT+solvent						
	B20						

Supplementary Figure 3 (*Continued*): (C) Isolated tumor-infiltrating leukocyte subpopulations of early-stage and end-stage tumors in young and old mice were analysed by multicolor flow cytometry. Statistics to frequencies of different myeloid and lymphoid subpopulations normalized to leukocytes. An upregulation of >10% is indicated in red, a downregulation of >10% is shown in blue and similar cell frequencies are marked in violet (*p<0.05, **p<0.01, ***p<0.01, unpaired Student's t-test, upper versus left condition).
